# Using lymph node transplantation as an approach to image cellular interactions between the skin and draining lymph nodes during parasitic infections^☆^^☆☆^

**DOI:** 10.1016/j.parint.2013.07.010

**Published:** 2014-02

**Authors:** Jennifer C. Lawton, Robert A. Benson, Paul Garside, James M. Brewer

**Affiliations:** Wellcome Trust Centre For Molecular Parasitology, Institute of Infection, Immunity & Inflammation, College of Medical, Veterinary & Life Sciences, University of Glasgow, Glasgow, G12 8TA, United Kingdom

**Keywords:** *In vivo* imaging, Microscopy, Immunology, Infection

## Abstract

The growing use of protozoan parasites expressing fluorescent reporter genes, together with advances in microscopy, is enabling visualisation of their behaviour and functions within the host from the very earliest stages of infection with previously unparalleled spatiotemporal resolution. These developments have begun to provide novel insights, which are informing our understanding of where host immune responses may be initiated, which cells are involved and the types of response that are elicited. Here we will review some of these recent observations that highlight the importance of cellular communication between the site of infection and the draining lymph node (dLN) in establishing infection and immunity. We also highlight a number of remaining challenges and unknowns that arise through our inability to follow and fate map the journey of a single cell between spatially separated tissue sites. In response to these challenges, we review a recently described experimental strategy that extends the spatial and temporal limits of previous imaging approaches, most significantly allowing longitudinal analysis of cellular migration between the skin and draining lymph nodes *in vivo*, without the requirement for invasive surgery.

## Highlights

• Role of skin and LN communication in establishing infection and immunity
• A technique to extend the spatial and temporal limits of tissue imaging
• This approach does not require invasive surgery for imaging.
• We can follow the journey of a single cell between spatially separated tissue sites.
• We outline future applications for this approach.

## Graphical abstract

**Figure:**
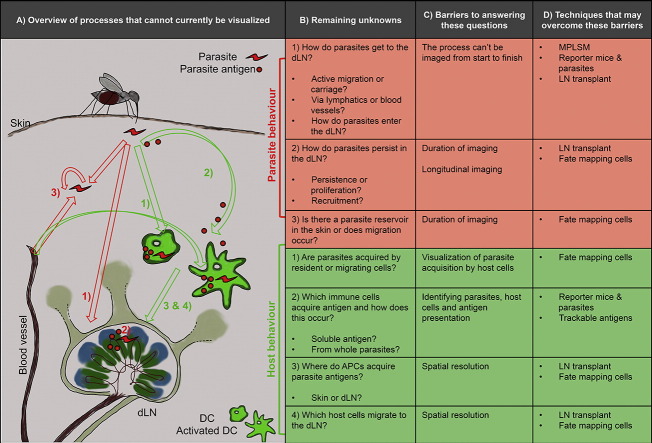

## 1 Introduction, Initiation of parasite infections by arthropod bite

Protozoan parasites such as *Plasmodium*, *Leishmania* and *Trypanosoma* spp. are transmitted by arthropod vectors that inoculate parasites into the skin during a blood meal. Diseases caused by these protozoa are responsible for at least 1.5 million deaths annually and considerable morbidity, particularly in tropical regions of the globe [1,2]. With the exception of cutaneous leishmaniasis, these are all systemic infections requiring metastasis of the initial skin infection to various affected organs via the peripheral circulation. Recent studies, particularly those applying *in vivo* microscopy have highlighted the previously unappreciated complexity of the early host/parasite interactions following deposition of protozoan parasites in the skin by vector feeding. This has been particularly well studied in murine models of malaria.

## 2 Murine malaria models as an example

Typically, around 50–150 *Plasmodium* sporozoites may be inoculated into the dermis from a single infectious mosquito bite [3,4]. A succession of papers have profitably applied intravital microscopy to image sporozoites in the skin of mice (reviewed by [5,6]). Briefly, these studies have shown that sporozoites are highly motile, with average velocities of 1–2 μm per second [7,8]. Their patterns of migration appear to be inherently random, with speed and/or direction restricted by the structure of the skin [9]. Although a role for chemotaxis has not been ruled out, it appears that the parasites do not actively migrate towards blood vessels [7,9]. In fact only a third of sporozoites appear to invade blood vessels, whilst approximately 15% can be found in lymphatic vessels [7]. Of the remaining 50% of the sporozoite bolus, more than a tenth appears to remain viable in the skin for at least 24 hours [10].

## 3 Infection and immunity in the skin and draining LN

Malaria parasites remaining in the skin following mosquito bite, have been shown to invade skin cells, where they commence development into exo-erythrocytic forms (EEFs). Parasite development has been observed to occur in the skin for several weeks (particularly in the immune-privileged hair follicles [10]), however merozoites released from these sites do not appear to be able to initiate a blood stream infection [11]. It has been postulated that this is due to a lack of motility by the merozoite stage of the parasite [5]. Gliding motility and the ability to traverse cells are required for sporozoites to migrate out of the skin [12,13]; the latter has been postulated to aid evasion of phagocytic cells until sporozoites can cross the endothelial barrier [13]. One consequence of cell traversal is that sporozoite antigens may be deposited in the cells they pass through. Additionally, parasite antigens are shed as sporozoites move, which could be picked up by local antigen presenting cells (APCs), or could drain directly to the dLN for acquisition by APCs there (reviewed by [5]).

As mentioned above, a significant proportion of parasites invade skin lymphatic vessels, some of which can be found in the dLN. Traditionally, it was believed that sporozoites entering the lymphatics would reach the peripheral circulation via the dLN and the thoracic or right lymphatic duct. However, the intravital imaging studies mentioned above demonstrated sporozoites internalised by, or in close contact with CD11c+ dendritic cells (DCs) [7]. Interestingly, EEFs could also be detected in podoplanin expressing cells within the dLN from 15 to 24 hours post infection, though not by 52 hours, suggesting that parasites were at least transiently present and viable in the dLN [7].

## 4 Induction of immune responses

Inflammation is one of the most immediate responses to injury or infection, recruiting immune effectors to the site by inducing vasodilation, increased capillary permeability and leukocyte extravasation into the tissue [14]. Vascular endothelial cells, platelets and innate tissue resident cells may produce small signalling molecules including cytokines and chemokines, which attract leukocytes to the site of infection. Subsequently, APCs such as DCs are able to act as a bridge between the innate inflammatory responses in the tissue site and adaptive branches of the immune response in secondary lymphoid organs. These functions include phagocytosis and sensing of invading pathogens through a variety of germline encoded receptors and consequently, activation of APCs that results in migration to the dLN, presentation of MHC/peptide complexes on their surface and expression of costimulatory molecules and cytokines required for T lymphocyte activation. Lymphocyte activation typically occurs in the lymph nodes, which collect interstitial fluid and cells draining from the tissue via lymphatic vessels. The cytokine and costimulatory environment in which priming occurs is influenced by the nature of the inflammatory response in the tissue and can induce different pathways of T cell activation [15]. Thus the immune system has to be equipped to transmit information on the very early interactions of invading microorganisms with host cells at the site of initial infection to subsequently influence subsequently generated immune responses in the dLN.

**Fig. 1:**
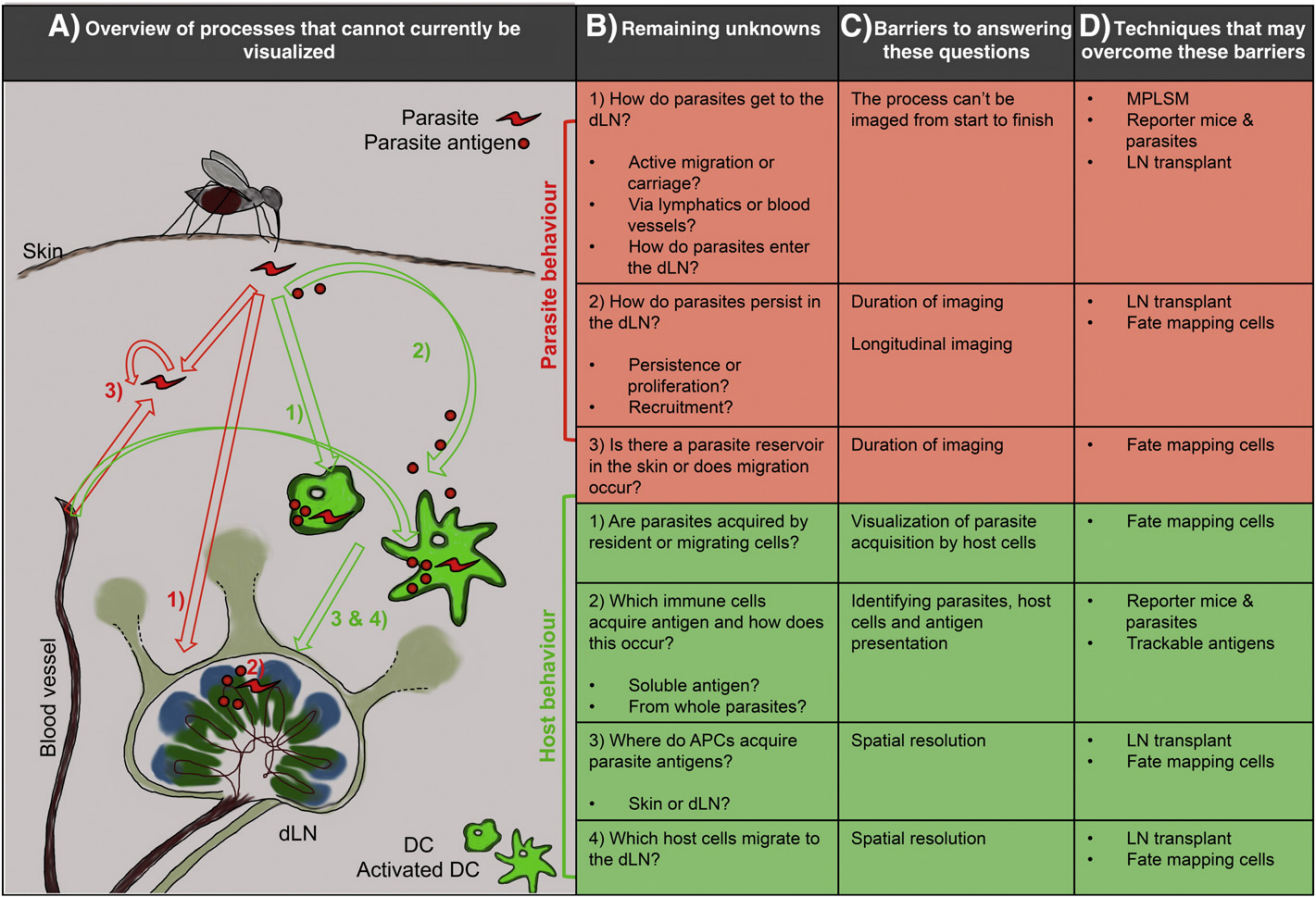
Outstanding questions during the initial stages of protozoan infection. Following inoculation into the skin after arthropod bite, gaps remain in our understanding of the interactions between parasites (red) and parasite antigen with host cells (green), such as DCs. In particular, it has been difficult to investigate parasite location and trafficking to the LN, via lymphatic and/or blood vessels, A). Major questions remaining about parasite (red) and host (green) behaviour are listed, B); depicted in A). In order to address these questions, several obstacles will need to be overcome, C); some possible solutions to these are listed, D).

The studies described above clearly demonstrate that the movement of immune cells or parasites between the initial infection site and the draining LN plays an important role in the establishment of infection and immunity in the host. Outstanding questions include how parasites, immune cells and antigens transit between these locations, the phenotype of these cells, and their positioning in tissues before and after migration; summarized in Fig. 1. In order to answer these questions, the ability to visualise the impact of skin infection upon the dLN will be crucial.

## 5 Visualisation of the dLN

To date, it has not been possible to image the processes of trafficking from infection site to the dLN in its entirety with cellular resolution, due to imaging limitations imposed by the spatial separation of these sites. Several groups have imaged excised dLNs (for example [16]), however this approach is unable to visualise migration of cells and parasites from the skin to the dLN or subsequent cellular recruitment to the dLN in real time. Imaging of explanted LNs is thus limited to visualization of cell behaviour within the LN.

Recently, methods for surgical exposure of the popliteal LN have been published which permit multi-photon imaging of the dLN after inoculation via the footpad [17,18]. This technique has yet to be applied to the study of endogenous cell behaviour and migration following parasitic infections. While this approach is more physiological than LN excision, the impact of the procedure itself remains an unknown. Surgery has the potential to impact on the local inflammatory environment and/or lymphatic flow. Critically, the feasibility of longitudinal study in the same animal remains limited (although not impossible). To circumvent such issues, our group has pioneered an alternative approach, which we describe below. Transplanting lymph nodes (tLNs) to the ear pinnae of mice allows direct imaging through the thin skin. By eliminating the need for surgical exposure to allow microscope access, the tissue can be imaged for longer in a single session and on consecutive days. Imaging the LN *in situ* also facilitates accurate study of antigen and cellular drainage as lymphatic vessels remain undisturbed. Thus by combining lymph node transplantation with the advantages of ear pinna imaging, tLNs represent skin draining lymph nodes, ideally suited to studying the interplay between tissue site and the dLN.

## 6 tLN analyses

The mouse ear lends itself well to non-invasive imaging of immune responses, having been used to study responses to injury, immunosurveillance and cutaneous infection [19–22]. The ears of anaesthetised mice can be held in isolation on a heated stage (necessary as decreases in temperature of the mouse extremities can be associated with some anaesthetics) and the animal placed directly under the microscope objective [23,24]. Importantly, as no invasive surgery is required to view the tissue and the method of restraint is mild, recovery of the animal for imaging at a later time (days or weeks later) makes longitudinal studies in the same animal possible [23]. In addition to being a readily accessible site for imaging studies, the mouse ear pinna is an amenable site for tissue transplant. Engrafted syngeneic cardiac tissue rapidly becomes vascularised, demonstrates electrical activity and can even be seen to pulsate [25–27]. Perhaps more relevant is the transplantation of spleen to the mouse ear pinna to facilitate successful intrasplenic immunisation for production of monoclonal antibodies. Neonatal spleens transplanted in this way retain red and white pulp, have normal T and B cell numbers, and, importantly, support induction of immune responses [42]. With transplant of lymphoid tissue a long-standing method to study immune processes *in vivo* [28–32], albeit often to less accessible sites such as the kidney capsule, transplant to the ear pinna has clear advantages.

Visualisation of cell behaviour in tLN through intact skin overcomes the requirement for removal or surgical exposure of the LN for imaging. Significantly, this has allowed the first longitudinal imaging of the same LN. The two key developments required to allow this were firstly, to establish the fidelity of the tLN as a *bona fide* lymphoid organ and secondly, to develop a microscope system capable of imaging at cellular resolution through the skin.

To establish that the transplant system faithfully creates a secondary lymphoid organ in the ear pinna we performed observational studies characterising the anatomical integrity, and cellular and molecular composition of tLN. The stromal cell network and blood and lymphatic supply within tLN appear similar to that observed in normal cervical LN. Further analysis revealed the presence of host and donor CD45^+^CD4^+^CD3^−^ LN inducer cells that are known to play a critical role in LN development through their interactions with stromal organiser cells, which induces the release of chemokines and growth factors [23]. Furthermore the presence of the chemokine CCL21, associated with coordinating localisation of CD4^+^ T cells could be detected in the paracortical regions. Within a tLN, the composition and positioning of key cell types required for the initiation of an adaptive immune response, including T cells, B cells and CD11c^+^ DCs were all comparable with a normal functioning LN. The presence of a vascular and lymphatic supply to the tLN could be demonstrated, and importantly these vessels were functional, capable of supporting lymphocyte recirculation and antigen drainage from tissue sites. Finally, administration of cognate antigen in tissue draining into the tLN induced antigen specific T cell activation and division demonstrating the tLN can function as a *bona fide* draining lymph node.

Imaging fluorescence signals in skin is problematic for a number of reasons. Key among these is the absorption of visible light by skin pigments, although other obstacles such as the inherent light scattering properties of tissue are an issue in intravital microscopy of skin and other organs. One approach to overcome the former problem has been to cross reporter strains of mice (usually on a C57BL/6 background) to albino strains (BALB/c, C57BL/6-C^2J^) [19,33]. Another approach is to use fluorescent reporters that are excited and/or emit in the infrared or near infrared spectrum (NIR). However excitation of these fluorochromes for Multiphoton Laser Scanning Microscopy (MPLSM) lies beyond the range of a conventional Titanium:Sapphire laser. To overcome these problems and allow deep imaging in the skin, we established a MPLSM system that combines a Titanium:Sapphire laser with an Optical Parametric Oscillator (OPO) [34], to generate a fully tuneable, two beam laser source, capable of two photon excitation of NIR probes. This approach could potentially be used to visualise T cells expressing the reporter dsRed [35] and DCs expressing YFP in the dLN without the requirement to surgically expose the tissue.

This novel approach to LN imaging brings a number of potential advantages. Firstly, we could measure the spontaneous 3D velocity of cells in the LN in the absence of removal or surgical exposure. These revealed a significantly lower mean T cell velocity (4 μm/minute) than we or others had previously reported in the literature using a variety of methods to reveal the LN [23]. Importantly, as surgery was not required to image the tLN, it was possible for the first time, to analyse whether surgery had any impact on the behaviour of cells in the LN. These investigations revealed an increase in velocity in tLN cells following sham surgery adjacent to the imaging area, suggesting a ‘Schrödinger's Cat’ situation where the response being observed is altered by the process of observation. While it could be argued that the slower velocity seen in tLN cells may be an artefact of the transplantation system, in the absence of an approach to directly image cells in the LN without the need for surgery we cannot answer this question. However, we can clearly conclude that surgery has a *de facto* impact on the cellular behaviour in the LN.

Importantly, the tLN approach can also allow longitudinal imaging of the same lymph node. Once imaging of the tLN has been performed the animal can be recovered and returned for imaging at various subsequent time-points. To allow identification of the same anatomical regions, we used intravenous injection of non-targeted quantum dots to allow blood vessels to act as fiduciary markers to re-register and orientate successive imaging fields. Previous intravital imaging studies in mice have typically been limited to a maximum of 6 hours due to ethical and scientific issues (for example artefacts induced by tissue/animal dehydration). Using the tLN system therefore expands the temporal dimensions available for cell imaging from hours to days, while maintaining resolution of minutes and seconds. In doing so, it is now possible to visualise multiple aspects relating to immune system function, allowing the study of developmental processes, interplay between spatially/temporally separated events and combination of different genetically modified tissues (see Table 1). Thus the tLN model fills the criteria needed to study immune responses incited in the skin, displaying normal function while being amenable to longitudinal non-invasive imaging with the LN and tissue site in close proximity.

## 7 Generating tLNs

Sex matched 4–5 week old syngeneic mice have been used as LN donors. LNs should be harvested in a laminar flow hood to promote the sterility of the donor tissue. Using appropriate dissection instruments, cervical LNs may be collected and placed straight into ice-cold PBS. These LNs would normally drain lymph from the mouse ear and most consistently provide successful grafts, however axillary and inguinal LNs have also been used successfully.

The area where the transplants themselves will be carried out should be washed down with 70% ethanol and covered with a sterile drape. Sterile instruments must be confined to clean areas. Recipient animals should then be weighed and anaesthetised using an injectable agent. Inhaled anaesthesia may also be used, however the presence of a mask can hinder access to the animal's head. For our studies, a combination of Hypnorm™ and Hypnovel™ has been used regularly, returning anaesthetised animals to a warmed cage (warm air circulation is preferable for recovery, however other sources such as heat mats would also be applicable). Hypnorm/Hynovel may be prepared as follows:

o Hypnorm™ (Vetapharma Ltd), Hypnovel™ (Roche) and ice cold sterile water should be mixed in the ratio of 1:1:6. This should be prepared fresh, to maximize efficacy.
o The anaesthetic mixture may be injected intra-peritoneally at a dose of 10 μL per gram of mouse body weight.
o This typically provides around 20 minutes of anaesthesia followed by sedation for approximately 4 hours (reviewed by [36]).

**Fig. 2:**
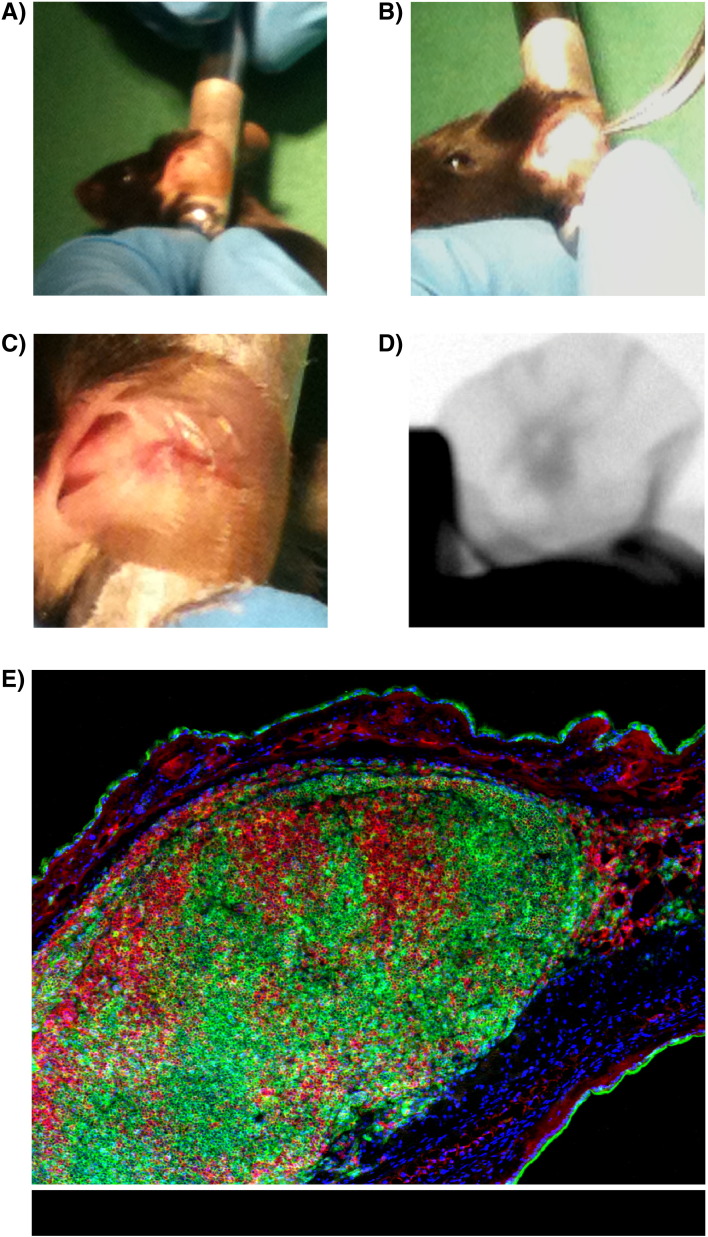
Implantation of a lymph node into the ear pinna. The process of LN implantation into the mouse ear pinna. The ear is secured and a pouch is formed, A); the LN is inserted into the pouch, B); and veterinary adhesive used to seal the opening, C). Implanted tissue may be vizualised by X-ray, D). An example section of an implanted LN, stained using DAPI, and antibodies recognizing B220 and CD4, E).

Double-sided adhesive tape may be wrapped around a cylinder, such as the metal spout of a drinking bottle (Fig. 2A). The mouse ear may then be immobilised onto this, acting as a stable platform for engrafting the new LN. The posterior of the ear pinna can then be adhered to the tape, rolling from the outer edge in toward the head. The point of a sharp pair of Dumont style forceps should be pressed through the dermis of the inner side of the ear (anterior) to produce a pocket into which the donor LN may be placed. Start closer to the outer edge of the ear, keeping the incision shallow while taking care not to puncture through the anterior surface again. Continue to press the closed forceps forward until the tips are in the centre of the ear. As the dermis is very thin here, the forceps should be clearly visible. Again, care should be taken to prevent puncture of the anterior pinna. A small sub-dermal pocket may be created by gently opening the tweezers (elastics can be wrapped around the tweezers to prevent them opening too far if desired).

The Dumont forceps may be left *in situ*, and the donor LN inserted deep into the pocket. This is most easily performed with a curved pair of forceps (Fig. 2B). Position the LN in the centre of the ear. The forceps are then carefully removed and the open end of the pocket flattened before sealing with a drop of veterinary grade tissue adhesive (Fig. 2C). The recipient animal may then be returned to the cage with a heat source until recovered. Transplanted LNs can be difficult to detect by eye, however, successful grafts may be visualised using whole body imaging technologies either employing detection of fluorescent signals from the graft or soft tissue X-rays (if the appropriate equipment is available) (Fig. 2D). Successful tLNs (Fig. 2E) are routinely analysed at three weeks post engraftment.

## 8 Conclusions

As discussed above, cellular communication between tissues and draining LNs has received considerable interest in the fields of immunology and infection, as well as in a range of other inflammatory and metastatic diseases. Intravital microscopy has added the temporal dimension to previous ‘snap shots’ of cells achieved using *ex vivo* approaches such as tissue histology or flow cytometry, however it has been impossible to fate map a single cell's life history in different tissues. This represents a significant limitation. For example, while it is believed that activation of DCs in tissues and their subsequent migration to the dLN is essential to the induction of primary T cell responses, whether a single cell performs all of these processes remains unclear as this process has never been observed from beginning to end *in vivo*.

It is equally unclear how parasites undergo dissemination following inoculation into the skin, such as the mechanisms by which they invade the afferent lymphatic vessels. These vessels are specialised in facilitating fluid and cellular transport from tissues, via specialised anatomical features such as discontinuous basal membranes surrounding the vessels and discontinuous tight junctions formed by lymphatic endothelial cells that produce ‘flaps’ in the vessel wall [37]. Whether invading parasites utilize these constitutive mechanisms, require induction of inflammation to facilitate opening of lymphatic flaps or rely on their own arsenal of proteolytic enzymes to invade lymphatics is unknown.

The tLN system allows a dLN to be situated close to the site of injection/infection, facilitating tracking of cell populations between these two sites. This would be particularly applicable in arthropod borne parasite infections such as trypanosomiasis, leishmaniasis or malaria, where metastasis of host cells and parasites from the initial bite site to the draining LN represents an important step in dissemination or host/pathogen interaction following infection. This technique in combination with optical highlighting tools allowing fate mapping of individual cells [38,39], tracking of antigen presentation [40], improved IR dyes for greater imaging depth and improved optics such as the mesolens [41], offers an exciting future in imaging disease processes from start to finish. Ultimately, such studies will inform our understanding of how particular interactions influence immunity to infection and will provide critical information about possible anti-parasite vaccination strategies.

## Figures and Tables

**Table 1:** Applications and benefits of the tLN model.

Developmental
• Lymphangiogenesis and vascularisation
• Memory/ageing
◦ Combine young LN stroma with aged mouse (and vice versa)
◦ Antigen experienced stroma and/or cells with naïve (and vice versa)
Visualising spatially or temporally separated events
• Longitudinal imaging
• Relocation to same area
• Physical movement of cells entering and leaving LNs
◦ Observation of injection site and draining LN synchronously
◦ e.g. Infections, vaccines, tumours
Enhancing the application of GM animals
• Labelling of cell subsets (compromised cells) in a normal context
• Generation of compound mutants/transgenics without complex breeding
◦ e.g. Cell specific knockouts (in transplants)
